# Rapid, Sensitive, and Accurate Evaluation of Drug Resistant Mutant (NS5A-Y93H) Strain Frequency in Genotype 1b HCV by Invader Assay

**DOI:** 10.1371/journal.pone.0130022

**Published:** 2015-06-17

**Authors:** Satoshi Yoshimi, Hidenori Ochi, Eisuke Murakami, Takuro Uchida, Hiromi Kan, Sakura Akamatsu, C. Nelson Hayes, Hiromi Abe, Daiki Miki, Nobuhiko Hiraga, Michio Imamura, Hiroshi Aikata, Kazuaki Chayama

**Affiliations:** 1 Department of Gastroenterology and Metabolism, Applied Life Sciences, Institute of Biomedical and Health Sciences, Hiroshima University, Hiroshima, Japan; 2 Laboratory for Digestive Diseases, Center for Genomic Medicine, RIKEN, Hiroshima, Japan; 3 Liver Research Project Center, Hiroshima University, Hiroshima, Japan; Saint Louis University, UNITED STATES

## Abstract

Daclatasvir and asunaprevir dual oral therapy is expected to achieve high sustained virological response (SVR) rates in patients with HCV genotype 1b infection. However, presence of the NS5A-Y93H substitution at baseline has been shown to be an independent predictor of treatment failure for this regimen. By using the Invader assay, we developed a system to rapidly and accurately detect the presence of mutant strains and evaluate the proportion of patients harboring a pre-treatment Y93H mutation. This assay system, consisting of nested PCR followed by Invader reaction with well-designed primers and probes, attained a high overall assay success rate of 98.9% among a total of 702 Japanese HCV genotype 1b patients. Even in serum samples with low HCV titers, more than half of the samples could be successfully assayed. Our assay system showed a better lower detection limit of Y93H proportion than using direct sequencing, and Y93H frequencies obtained by this method correlated well with those of deep-sequencing analysis (r = 0.85, P <0.001). The proportion of the patients with the mutant strain estimated by this assay was 23.6% (164/694). Interestingly, patients with the Y93H mutant strain showed significantly lower ALT levels (p=8.8 x 10^-4^), higher serum HCV RNA levels (p=4.3 x 10^-7^), and lower HCC risk (p=6.9 x 10^-3^) than those with the wild type strain. Because the method is both sensitive and rapid, the NS5A-Y93H mutant strain detection system established in this study may provide important pre-treatment information valuable not only for treatment decisions but also for prediction of disease progression in HCV genotype 1b patients.

## Introduction

Hepatitis C virus (HCV) is a major cause of chronic liver disease, liver cirrhosis, and hepatocellular carcinoma, affecting up to 180 million people worldwide [1,2]. Dual oral treatment with the NS5A inhibitor daclatasvir (DCV) and the NS3 protease inhibitor asunaprevir (ASV) was one of the first interferon (IFN)-free regimens examined in treatment-experienced patients with genotype 1 HCV infection. HCV often acquires resistance against direct acting antiviral agents (DAAs) [3]. Presence of the Y93H mutation prior to treatment has been reported as an important predictor of virologic failure [4,5,6,7]. The pre-existing Y93H mutation has been estimated by direct sequencing to be present in 8.3%–19% of Japanese patients [8,9].

Direct sequencing is commonly used to detect viral mutations. However, it is only capable of detecting viral subpopulations with frequencies no lower than 10% to 20% [10,11,12,13]. In contrast, next generation sequencing (NGS), which has recently been applied to analyze viral mutations, can detect relatively low frequency variants (≤ 1%) [14,15], but it is still complex to perform and prohibitively expensive for widespread clinical use.

The Invader assay is better suited for high-throughput SNP typing [16]. To take advantage of its specificity and quantitative nature, the Invader assay has also been used for analysis of allele-specific transcription [17], detection of copy number variation [18] and drug-resistant hepatitis B virus variants [19].

In this study, we developed a rapid NS5A-Y93H strain detection system based on the Invader assay to evaluate the proportion of HCV genotype 1b patients with pre-existing Y93H mutations.

## Materials and Methods

### Study subjects

A total of 702 serum samples of Japanese HCV genotype 1b infected patients were screened in the study. All patients were NS5A inhibitor-treatment-naïve chronic hepatitis C patients with genotype 1b. Serum HCV RNA was measured at a central laboratory using the Roche COBAS TaqMan HCV Auto assay (Roche Diagnostics K.K., Tokyo, Japan). HCV genotype was determined at the central laboratory by polymerase chain reaction (PCR) amplification and direct sequencing. The study was approved a priori by the ethical committee of Hiroshima University and conforms to the ethical guidelines of the 1975 Declaration of Helsinki. All patients provided written informed consent.

### HCV RNA extraction and cDNA synthesis

Total RNA was extracted from 150 μL of each serum sample using NucleoSpin RNA virus columns (Macherey-Nagel, Düren, Germany) according to the manufacturer's instructions. cDNA was synthesized using the PrimeScript RT reagent Kit with gDNA Eraser and oligo dT primer (TaKaRa, Otsu, Shiga, Japan).

### Nested-PCR

**Fig 1:**
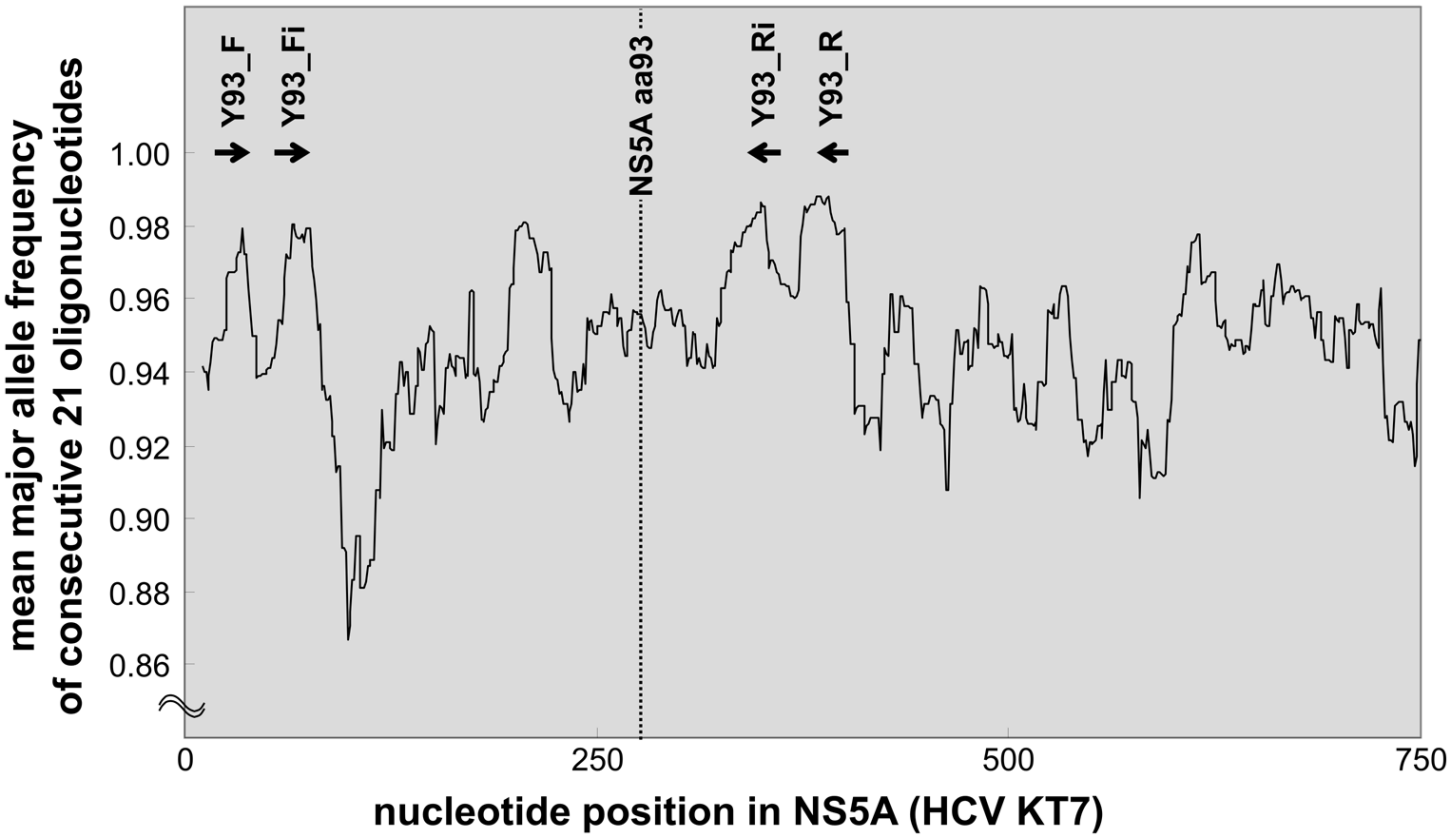
Major base frequencies of consecutive 21 nucleotides in the NS5A region. Using 240 HCV-1b NS5A sequence data from a publicly-available HCV database, mean major base frequency of any 21 bp nucleotide sequences located at every positions are plotted. A higher major base frequency was assumed to represent lower variability. Finally, two degenerate primer sets for nested PCR were designed at positions shown with bold arrows, which contained alternative bases at several polymorphic sites to increase PCR success rate.

**Fig 2:**
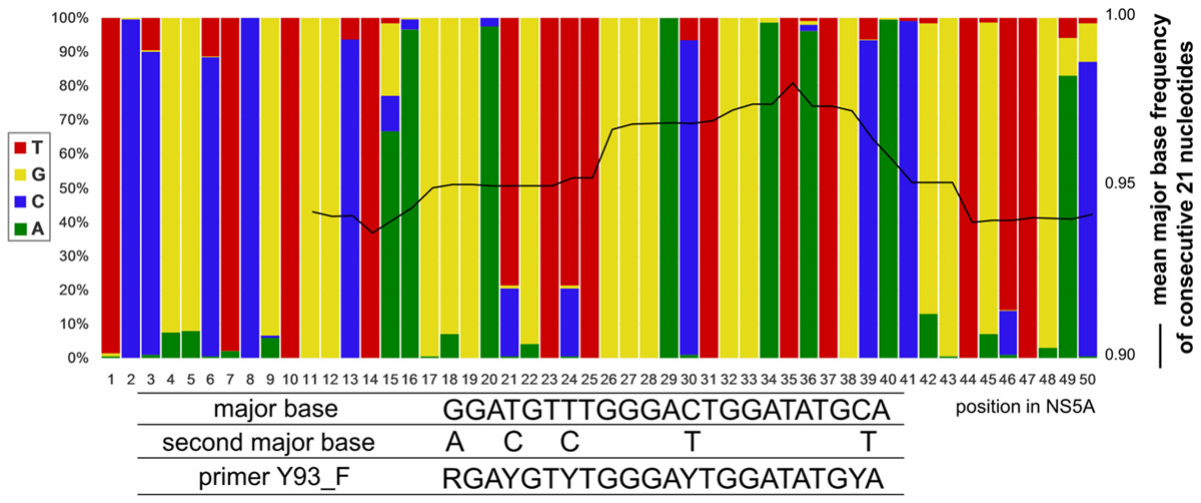
An example of designing process of degenerate primers and probes. The stacked bar represents the proportion of each base at a given nucleotide position in the NS5A region of HCV-KT9 calculated from 240 NS5A sequences of HCV genotype 1b. The solid black line represents mean major base frequency of consecutive 21 nucleotides. The Y93_F primer was placed where overall mean major base frequency can be approximately maximized, and thereafter alternative bases were included at several positions to avoid mismatch with minor base substitutions. A trade-off relationship between specificity and coverage should be considered. Basically, the same holds for the design for Invader probe set, except that there is less positional flexibility from the nature of the principle itself.

When designing PCR primers and Invader probes, a total of 240 NS5A sequences of HCV genotype 1b from a publicly-available database [20] were utilized as a guide for successful primer and probe design. All sequences were aligned using the CLUSTALW program. The major base frequency in each nucleotide position, and thereafter, their average in each consecutive 21-bp window, was calculated and plotted (Fig 1). A higher mean major base frequency was assumed to represent lower variability at a given position and presumably improve its suitability for inclusion in a PCR primer. We used degenerate primers (Table 1), which contain alternative bases at several polymorphic sites. An example of the designing process of degenerate primer is shown in Fig 2. A fragment of 308 bp within the NS5A region was amplified from cDNA by nested PCR. The thermal profile of the initial PCR was 95°C for 2 min, followed by 35 cycles at 95°C for 15 s, at 60°C for 45 s, and at 72°C for 60 s. An aliquot of the PCR product (5 μl) was used in the nested PCR. The thermal profile for the nested PCR was the same as for the initial one, except the number of cycles was changed to 20. Amplification products were checked by agarose gel electrophoresis.

**Table 1:** Sets of nested PCR, Invader probes, and synthesized standard oligonucleotides.

	name	nucleotide sequences (5'-3')	nucleotide position for the NS5A region^a^
**outer primers**			
	Y93_F	rgaygtytgggaytggatatgya	18–40
	Y93_R	yggtcatgccygtyacrtart	383–403
**inner primers**			
	Y93_Fi	ttcaaracytggctycrrtcya	55–76
	Y93_Ri	ayctccacgtaytcctcrgcrg	341–362
**invader probe set**			
	Invader oligo	gtgcaggggccccgtggtrtt	271–290
	Y93 wild probe^b^	CGCGCCGAGGahgcgttratgggraayg	254–271
	Y93 mutant probe^c^	ATGACGTGGCAGACghgcgttratgggraay	255–271
**synthesized standard nucleotides**			
	std_YY	ttcaagacctggctccagtccagtgcaggggccccgtggtgtatgcgttgatggggaatggtgcaggggccccgtggtgtatgcgttgatggggaatgctgctgaggagtacgtggaggt	
	std_YH	ttcaagacctggctccagtccagtgcaggggccccgtggtgtatgcgttgatggggaatggtgcaggggccccgtggtgtgtgcgttgatggggaatgctgctgaggagtacgtggaggt	
	std_HH	ttcaagacctggctccagtccagtgcaggggccccgtggtgtgtgcgttgatggggaatggtgcaggggccccgtggtgtgtgcgttgatggggaatgctgctgaggagtacgtggaggt	

Uppercase letters represent the 5' flap of probe.

^a^HCV-KT9 (accession number AB435162) was used as a reference.

^b^linked with FRET probe including Yakima-Yellow (Y-Y) as fluorescent reporter.

^c^linked with FRET probe including 6-carboxyfluorescein amino hexy (FAM) as fluorescent reporter.

### Invader assay

**Fig 3:**
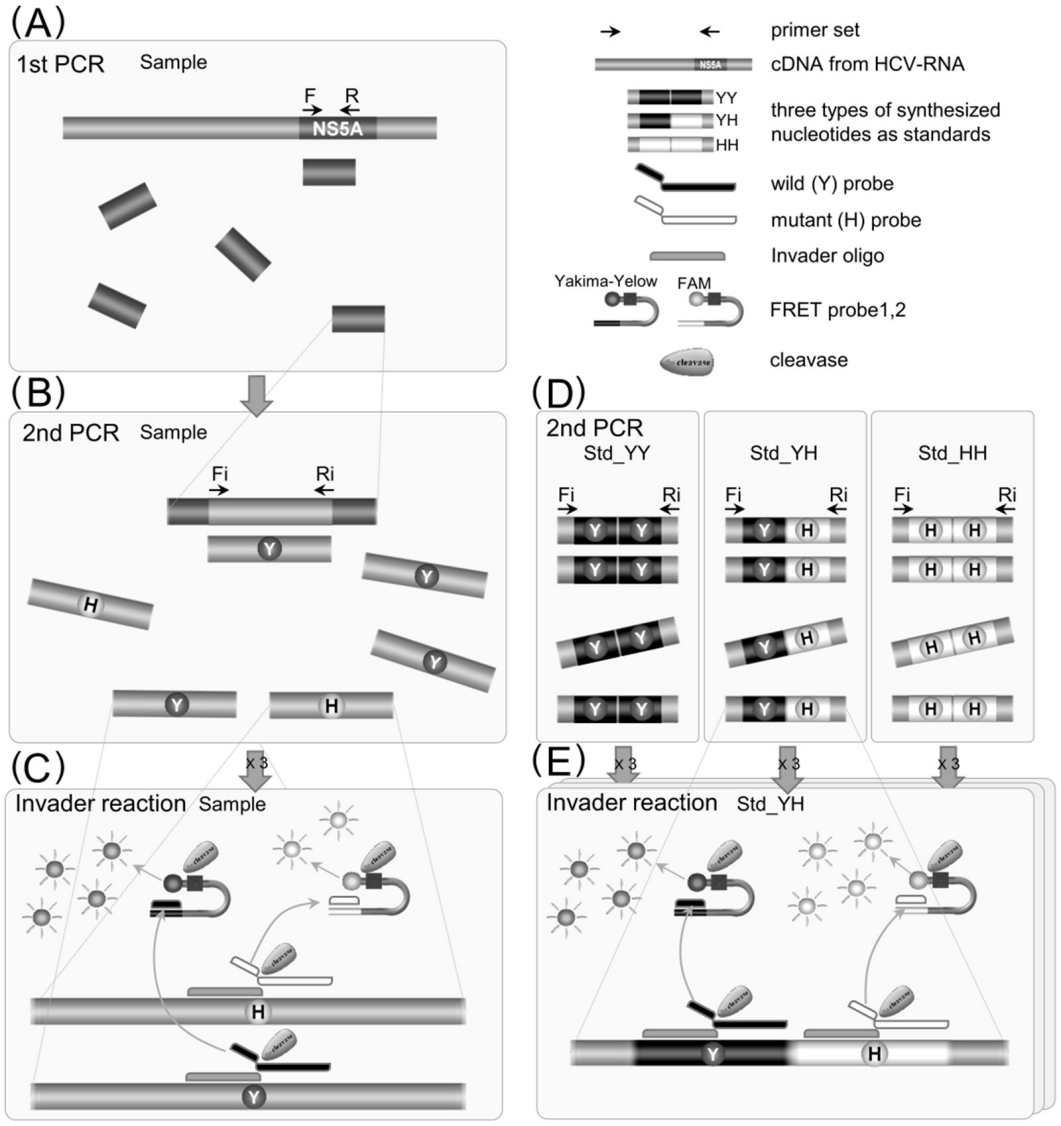
Schematic flow diagram representing a method of nested-PCR followed by Invader assay. (A) The initial PCR was performed to amplify a fragment of 386 bp length containing a part of the NS5A region from cDNA reverse-transcribed from HCV-RNA which was extracted from the serum of a patient. (B) An aliquot of the inital PCR product was used for the second PCR to amplify a 308 bp fragment. The second PCR product was diluted with water and subjected to the Invader assay. (C) Invader oligonucleotide and allele-specific probes anneal with target to form one base overlap. When the base is complementary to the opposing base in the allele-specific probe, cleavase recognizes the structure and releases 5’ flap. The released flap anneals to a FRET probe. The second cleavage reaction releases fluorophore resulting in the generation of a fluorescent signal. The Invader assay was done in triplicate. (D) Three types of standard nucleotide (Std-YY, Std-YH, and Std-HH) were prepared, which includes binary target sequences, corresponding to wild (Y) or mutant (H) variants, and annealing sites with the internal primers at each end. Tenfold serial dilusions of each standard were subjected to the second PCR. (E) The Invader assay for each standard was also performed in triplicate.

Three nucleotide sequences, which also contained several degenerate sites, were designed for the Invader assay (Table 1). For the calibration of the proportion of mutant variants, three types of standard nucleotide were prepared: Std-YY, Std-YH, and Std-HH (Table 1). Each sequence includes binary target sequences, corresponding to wild (Y) or mutant (H) variants, and also includes annealing sites with the internal primers at each end. An additional four nucleotides were inserted between the binary target sequences to avoid interference. Dilution series of these standards were assayed in triplicate with each assay. For Invader assays, 384-well reaction plates were used with the ABI Prism 7900 HT sequence detection system (Life Technologies, Foster City, CA, USA). The plate was incubated isothermally at 63°C and fluorescence intensities were detected every 2 min for 40 min or until non-specific fluorescence [21] was detected. Each sample was tested in triplicate in the same plate. A flow diagram representing a method of nested-PCR followed by Invader assay is depicted in Fig 3.

### Deep sequencing

The frequency of NS5A-Y93H was determined by ultra-deep sequencing using the Ion Torrent PGM (Life Technologies) according to previously described protocols [22] with some modifications. In brief, the fragment distributions of all amplicons were analyzed on a Bio-Analyzer 2100 (Agilent Technologies, Palo Alto, CA). The amplified fragments were modified by the Ion Xpress Plus Fragment Library Kit, and sequence analysis was performed using Ion PGM. Read mapping onto the HCV-KT9 reference sequence (GenBank accession no. AB435162) was performed using the CLC Genomics Workbench software (CLC bio, Aarhus, Denmark). This technique revealed an average coverage depth of over 10,000 reads per position in the unique regions of the genome. A wild type hepatitis C virus-expressing plasmid pHCV-KT9 [23] was used to estimate the minimum detection threshold of Y93H frequency. The sequence data have been deposited in the NCBI Short Read Archive under BioProject PRJNA275480.

### Statistical analysis

For general statistical analysis, we employed the R statistical package. Student's t-test or chi-square test were used as appropriate. All statistical analyses were 2 sided, and P < 0.05 was considered significant.

## Results

### Estimation of Y93H mutant proportion by standard oligonucleotides

**Fig 4:**
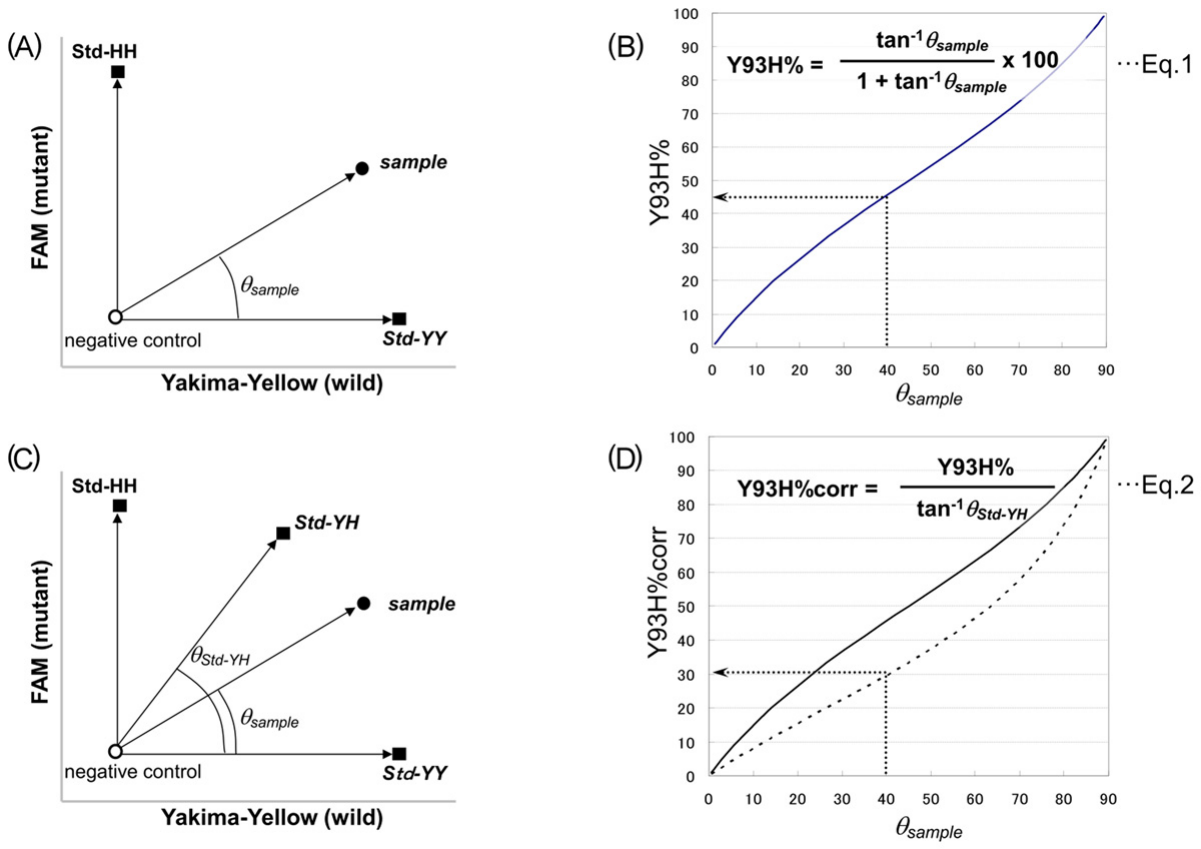
Schematic principle for the estimation of Y93H proportion by Invader assay. **(A)** Observed fluorescence intensities of samples and standards were plotted on a two-dimensional graph, where the X-axis was Yakima-Yellow intensity and the Y-axis was FAM intensity, corresponding to Y93 wild and Y93H mutant strains, respectively. By using crossing angle *θ*_*sample*_, the Y93H mutant proportion of a sample can be calculated with Equation 1. **(B)** The resulting curve of *θ*_*sample*_ vs Y93H% is nearly linear. **(C)** Although the PCR product from the Std-YH theoretically contains equal amounts of Y93 wild type and Y93H mutant type target sequences, the plot of the Std-YH did not always head in the intermediate direction between Std-YY and Std-HH. **(D)** Therefore, by using a crossing angle *θ*_*Std-YH*_, The corrected Y93H mutant proportion Y93H%corr can be calculated from Y93H% with Equation 2. Estimation was carried out using data at an appropriate time point before the emergence of non-specific fluorescence.

After nested PCR, the products were diluted up to 20-fold and used as templates for the Invader assay. Fluorescence intensities were measured intermittently up to 40 minutes of incubation or until emergence of non-specific fluorescence. A two-dimensional plot was made based on the relative fluorescence signal intensities of the two probes (Yakima-Yellow/Rox and FAM/Rox). As shown in Fig 4A, by using a crossing angle *θ*_*sample*_ between the Std-YY and given sample, the Y93H mutant proportion can be calculated with Equation 1, which is represented by the nearly linear curve of *θ*_*sample*_ vs Y93H% in Fig 4B. Notably, the Std-YH did not always head in the intermediate direction between Std-YY and Std-HH (Fig 4C), showing that the same intensities of the two kinds of fluorophore signals do not necessarily correspond to an equivalent amount of Y93 wild and Y93H mutant strains. By using a crossing angle *θ*_*Std-YH*_ between the Std-YY and the Std-YH, A corrected Y93H mutant proportion, Y93H%corr, can be derived from Y93H% with Equation 2 as shown in Fig 4D. Estimation was carried out using data at an appropriate time point before the emergence of non-specific fluorescence.

### HCV RNA level and assay success rate

**Fig 5:**
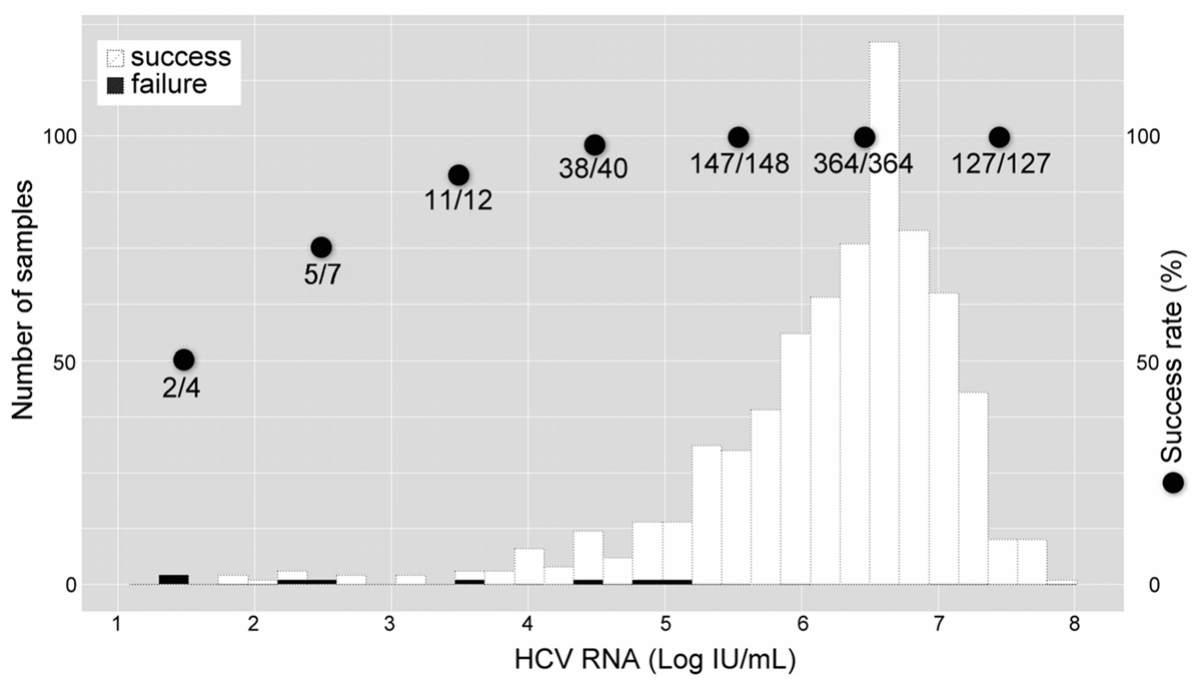
Histogram of HCV RNA levels according to the detectability of Y93H by the Invader assay. White and black bars represent successful and unsuccessful detection cases, respectively. Closed circles represent the success rate for each log level of viremia from 1 through 7.

Next, the assay success rate and the lower detection limit in HCV RNA level were evaluated. Fig 5 shows histograms of successful samples and unsuccessful samples based on their HCV titer assayed by a quantitative commercial RT-PCR assay. The overall success rate was 98.9% (694/702). Even in serum samples with low HCV titers, more than half of samples could be successfully assayed. Thus, the lower detection limit of HCV RNA level could be estimated to be almost 1 log order. Among 694 patients successfully tested, the mutant strain (Y93H) was observed in 23.6% (164/694) patients.

### Sensitivity of the Invader assay for NS5A-Y93H strain detection

**Fig 6:**
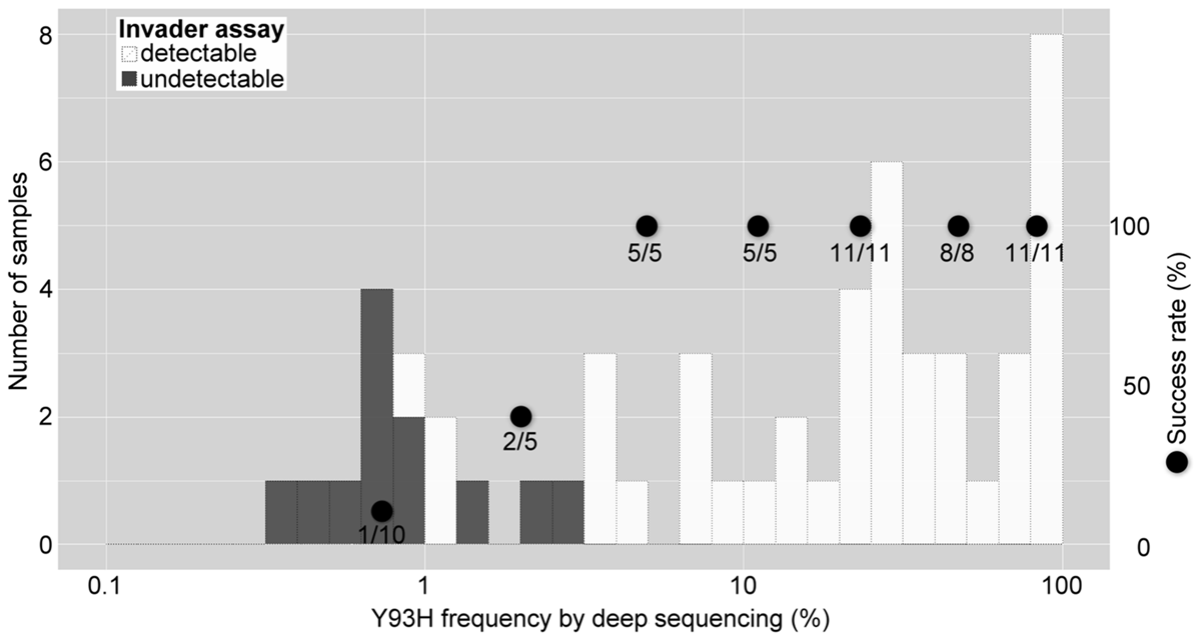
Histogram of Y93H frequency by deep sequencing according to detectability by the Invader assay. Deep-sequencing was performed using 55 sera of HCV 1b patients with various Y93H frequencies by the Invader assay. White and black bars represent successful detection cases and detection failure cases, respectively. Closed circles represent the success rate.

Sensitivity for NS5A-Y93H strain detection was evaluated in comparison with the deep-sequencing data of 55 sera of HCV 1b patients. These were selected to include various proportions of NS5A-Y93H strain by this assay. As shown in Fig 6, among samples containing more than 2.8% mutant, detection of Y93H mutant variant by the Invader assay was consistently successful, and the lower detection limit of the proportion of Y93H strains could be estimated at 1 to 2%.

### Correlation for measurement of Y93H proportion

**Fig 7:**
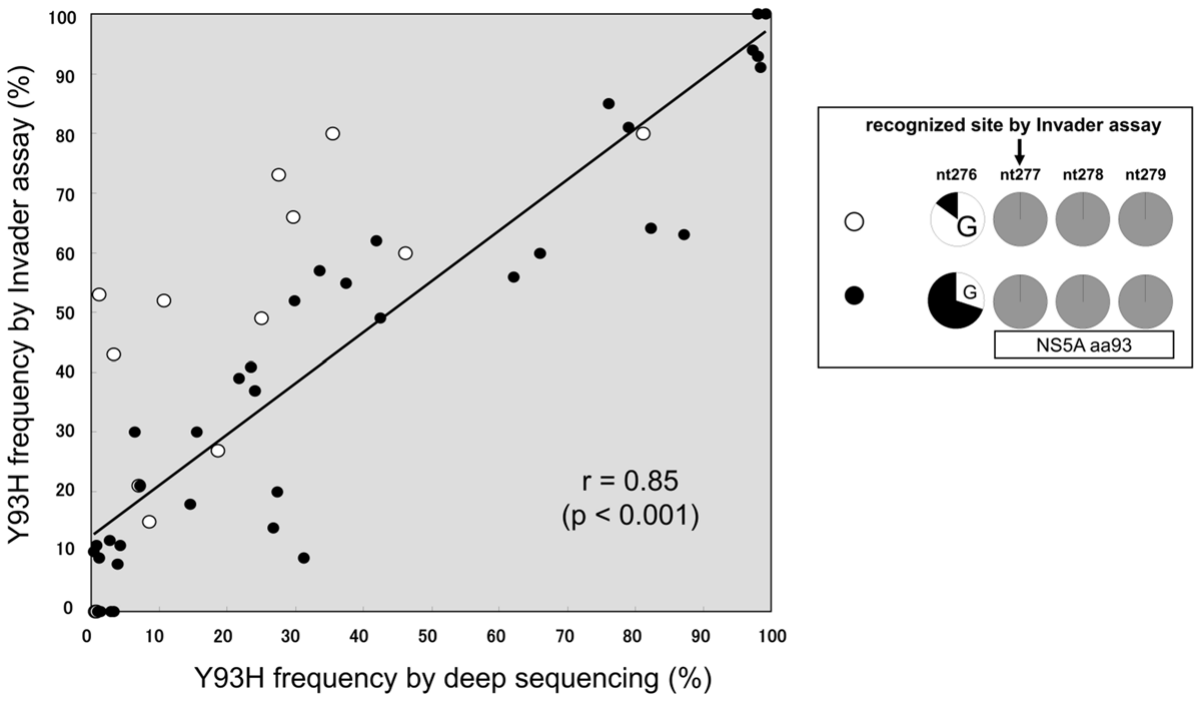
Correlation between deep-sequencing and Invader assay on the measurement of Y93H frequency. Positive correlation was observed for the Y93H strain frequencies between deep-sequencing and the Invader assay of 55 HCV 1b patients (Spearman correlation r = 0.85, p < 0.001). In samples in which G allele at the preceding nucleotide position before NS5A amino acid 93 was predominant (> 85%) measured by NGS (open circle), there was a tendency to overestimate the Y93H proportion by the Invader assay.

A significant positive correlation was observed between the proportion of Y93H strains determined by this assay system and the proportion determined by NGS (r = 0.85, P <0.001) (Fig 7). However, in samples in which a G allele was predominant at the preceding nucleotide position adjacent to NS5A amino acid 93 (plotted by open circles), there was a tendency to overestimate the Y93H proportion by Invader assay.

### Association between clinical phenotypes and Y93H

We investigated possible relationships between several clinical phenotypes and Y93H strains. As shown in Table 2, patients with the Y93H mutant strain showed significantly lower ALT levels (p = 8.8 x 10^–4^), higher serum HCV RNA levels (p = 4.3 x 10^–7^), and lower HCC risk (p = 6.9 x 10^–3^) than those with the wild type strain.

**Table 2:** Clinical characteristics of 702 patients infected with HCV genotype 1b included in the study.

		Invader assay		P value^a^
	Total (n = 702)	Y93 wild (n = 529)	Y93H mutant (n = 164)	(wild vs mutant)
Age	66.5±11.4	66.1±11.4	67.8±11.5	0.12
Gender (Male:Female)	296:406	229:300	59:105	0.097
Aspartate aminotransferase, IU/L	56.1±37.0	58.6±39.8	47.6±23.6	0.12
Alanine aminotransferase, IU/L	60.5±45.0	65.1±48.3	44.4±25.2	8.8 x 10^−4^
γGTP, IU/L	61.2±57.4	62.5±61.4	57.7±41.7	0.59
Hemoglobin, g/dL	13.5±1.8	13.6±1.6	13.1±2.49	0.56
White blood cells count, cells/mm3	4.97±1.54	4.94±1.54	5.03±1.57	0.74
Platelet count, x 104 cells/mm3	15.0±6.1	14.8±6.2	15.5±5.7	0.39
HCC (+:-)	142:560	119:410	21:143	6.9 x 10^−3^
HCV RNA, log IU/ml	6.20±0.95	6.14±0.89	6.53±0.79	4.3 x 10^−7^
past IFN therapy (Naive:Experienced)	363:312	269:247	91:61	0.093

^a^ Data were compared by Student's t-test or chi-square test as appropriate.

## Discussion

Several mutations in HCV structural and non-structural proteins have been reported to be associated with interferon resistance, including Core [24], NS3/NS4A, NS5A [25,26], and E2 [27]. In all classes of DAAs, rapid selection of resistant variants and viral breakthrough have also been observed [3]. Such resistant variants can arise from pre-existing viral subpopulations in the patient [28]. Several DAA combination therapies, with or without pegylated-IFN plus ribavirin, were reported to minimize the emergence of resistance and improve efficacy. Dual oral therapy with ASN and DCV was one of the first interferon-free regimens to undergo clinical evaluation and has recently been approved for use in Japan. ASN and DCV therapy results in a high rate (65%–78%) of sustained virological response (SVR) in genotype 1b patients who were null responders during prior interferon therapy; however, the Y93 mutation has been reported to be an independent predictor for non-SVR with DCV plus ASV therapy by multivariate analysis [6].

Under such circumstances, we attempted to establish a rapid, sensitive, and cost-effective method to detect the presence of the drug resistant NS5A-Y93H strain to inform treatment decisions in a practical clinical setting. HCV exists as a highly heterogeneous population of so-called quasispecies [29]. To avoid the adverse effects of hypervariability of the HCV genome on the assay, PCR-priming sites were selected based on sequence analysis of 240 HCV genotype 1b NS5A sequences from the database. To compensate for common sequence variants, several degenerate sites were included in the PCR primers and Invader probes to improve the success rate. A high overall success rate of 98.9% (694/702) was attained, which was higher than that of the earlier method for Y93H detection by real-time PCR using cycling probes [30]. In order to increase successful detection in samples with low HCV titer, nested PCR was undertaken to reach saturation in as many samples as possible. This assay protocol was oriented to evaluate the relative amount of Y93H mutant strain compared to wild strain but not to evaluate the quantity of both strains because serum HCV RNA levels can be easily quantified by commercially available assays.

Deep sequencing is increasingly being utilized to detect low frequency drug resistant HCV variants. The reported error rate of NGS platforms ranges from a few tenths of a percent to several percent [31]. We have previously reported that the minimum Y93H variant frequency detection threshold for deep-sequencing was 0.3% and that Y93H variants could be detected in 50% (5/10) patients before DCV treatment [32]. Miura et al reported that Y93H mutations with a frequency of 1% or higher could be detected by deep-sequencing. In larger studies by deep-sequencing, higher prevalence rates (25%–30.9%) of Y93H were reported [10,33], which were higher than that of direct sequencing (8.2%–19.0%) [8,9]. In this study, our assay system achieved a better lower detection limit of Y93H, estimated to be 1 to 2%, than direct sequencing, although it is not as sensitive as deep-sequencing. The prevalence rate (23.6%) of Y93H estimated by our assay is comparable with that assayed by real-time PCR using cycling probes (19.7%)[30], and ranked between those of deep sequencing and direct sequencing reported in Japanese population, presumably reflecting the degree of lower detection limit of Y93H. Natural prevalence of NS5A Y93H in Japanese population seems to be comparable with those in Western and South American countries examined by direct sequencing [34,35,36].

There were eight samples which failed in this assay, including four of HCV RNA levels within detection limit of the assay. Amplified products were not detected in all these samples by gel electrophoresis. Then we performed nested PCR using two sets of primers designed within a highly conserved 5' untranslated region [37]. Successful amplification was achieved in three samples of relatively high viral titer. Redesign of the primers may improve success rate. In addition to HCV RNA levels, the assay success may be possibly affected by degradation of viral RNA during several freeze/thawing cycles during the long-term storage.

A significant positive correlation was observed between the Invader assay and NGS with respect to Y93H proportion (Fig 7). However, a tendency of overestimation was observed in samples in which a G allele was predominant at the preceding nucleotide (nt276) in the codon adjacent to NS5A aa 93.

We also investigated possible relationships between several clinical phenotypes and Y93H and found that the existence of the Y93H mutant strain was significantly associated with ALT level, serum HCV titer, and HCC risk. Our data show similarities (HCV RNA) and differences (age, ALT, and platelet counts) when compared with those reported by Itakura et al [8], although they were analyzed by direct sequencing. Interestingly, association between Y93H and favorable *IL28B*/*IFNL4* genotype has been recently reported by our group and others in Japan [8,33,38]. *IL28B*/*IFNL4* variant influences viral response to interferon-based HCV therapy as well as persistent HCV infection [39,40]. These findings suggest that Y93H mutation not only confer resistance to NS5A inhibitors but also interact with genes involved in innate immunity and pro-inflammatory response such as *IL28B* [8,33,38]. Further research with larger samples and functional analysis is needed.

In conclusion, we developed a rapid detection system for estimating NS5A-Y93H strain frequency. This system attained high assay success rates, and sensitive detection of Y93H compared to direct sequencing. The information of NS5A-Y93H strain may provide important information not only for treatment decisions but also for prediction of disease progression in HCV genotype 1b patients.
